# Pathogen species are the risk factors for postoperative infection of patients with transurethral resection of the prostate: a retrospective study

**DOI:** 10.1038/s41598-023-47773-7

**Published:** 2023-11-28

**Authors:** Jiexiang Lin, Zesong Yang, Liefu Ye, Yun Hong, Wanghai Cai, Honghong Pan, Haishou Fu, Jinfeng Wu

**Affiliations:** 1 The Shengli Clinical Medical College, Fujian Medical University, Fuzhou, 350001 Fujian China; 2 Department of Urology, Shengli Clinical Medical College of Fujian Medical University, Fujian Provincial Hospital, Fuzhou, 350001 Fujian China; 3 Department of Clinical Laboratory, Shengli Clinical Medical College of Fujian Medical University, Fujian Provincial Hospital, Fuzhou, 350001 Fujian China

**Keywords:** Diseases, Risk factors, Urology

## Abstract

This study aimed to analyze the infection risk factors for transurethral resection of the prostate (TURP) and establish predictive models to help make personalized treatment plans. Our study was designed one-center and retrospectively enrolled 1169 benign prostatic hyperplasia (BPH) patients. Risk factors were explored for postoperative infection. A TURP-postoperative infection (TURP-PI) model with infection prediction values was created. The improved-TURP-PI (I-TURP-PI) model, including extra new factors (pathogens species), was also built to see whether it could optimize the prediction abilities. At last, we developed a nomogram for better clinical application. Operation time, preoperative indwelling urinary catheter (PIUC), and positive preoperative urine culture were independent risk factors (all *P* < 0.05). Interestingly, pathogens species in pre-surgery urine (*P*_Enterococcus faecium_ = 0.014, *P*_Pseudomonas aeruginosa_ = 0.086) were also independent risk factors. Patients with positive Enterococcus faecium (37.50%) were most likely to have postoperative infection. We built two models with AUC_TURP-PI_ = 0.709 (95% CI 0.656–0.763) and AUC_I-TURP-PI_ = 0.705 (95% CI 0.650–0.760). The nomogram could help improve the prediction ability. To our knowledge, our study is the first to use pathogen species in urine before surgery as risk factors for infection prediction after TURP. TURP-PI and I-TURP-PI models have essential roles in predicting patients' postoperative infections and in better postoperative antibiotic decision-making.

## Introduction

Benign prostatic hyperplasia (BPH) is a common condition that could affect the life quality of a man over 50. Roughly half of all men will suffer from BPH-related symptoms later in life^1,2^. Transurethral resection of the prostate (TURP) is a gold-standard surgery procedure for BPH patients ^3^. Although TURP is generally considered safe, effective, and well-tolerated, it is also associated with numerous complications, including infection, transurethral resection syndrome, bleeding, and prolonged hospitalization^4,5^. Studies showed that the infection rate after TURP ranged from 2.6 to 13.5% ^6–9^. A postoperative infection will prolong hospital stay, increase medical expenses, and affect patients' quality of life. In addition, there will be a 0.7–4.4% chance of progressing to sepsis, which could endanger patients' life ^8–10^. Studies showed that postoperative infection was related to factors such as positive preoperative urine culture, the preoperative indwelling urinary catheter (PIUC), duration of operation time, diabetes, age, use of prostate-related drugs, preoperative nutritional status, and antibiotics usage^8,11,12^. Elevated prostate-specific antigen (PSA) level may also be indicative of undetected infections in BHP patients ^13^.

As far as we know, there may not be comprehensive guidelines on postoperative infection prediction of TURP from the European Association of Urology (EAU) or the American Urological Association (AUA). Also, there is a lack of high-risk pathogen species and predictive models for postoperative infection after TURP in the literature. This study aimed to analyze the risk factors of infection susceptibility for BHP patients with TURP and establish warning scoring models to provide a basis for preventing and treating postoperative urinary infections.

## Results

### Clinical characteristics

**Figure 1:**
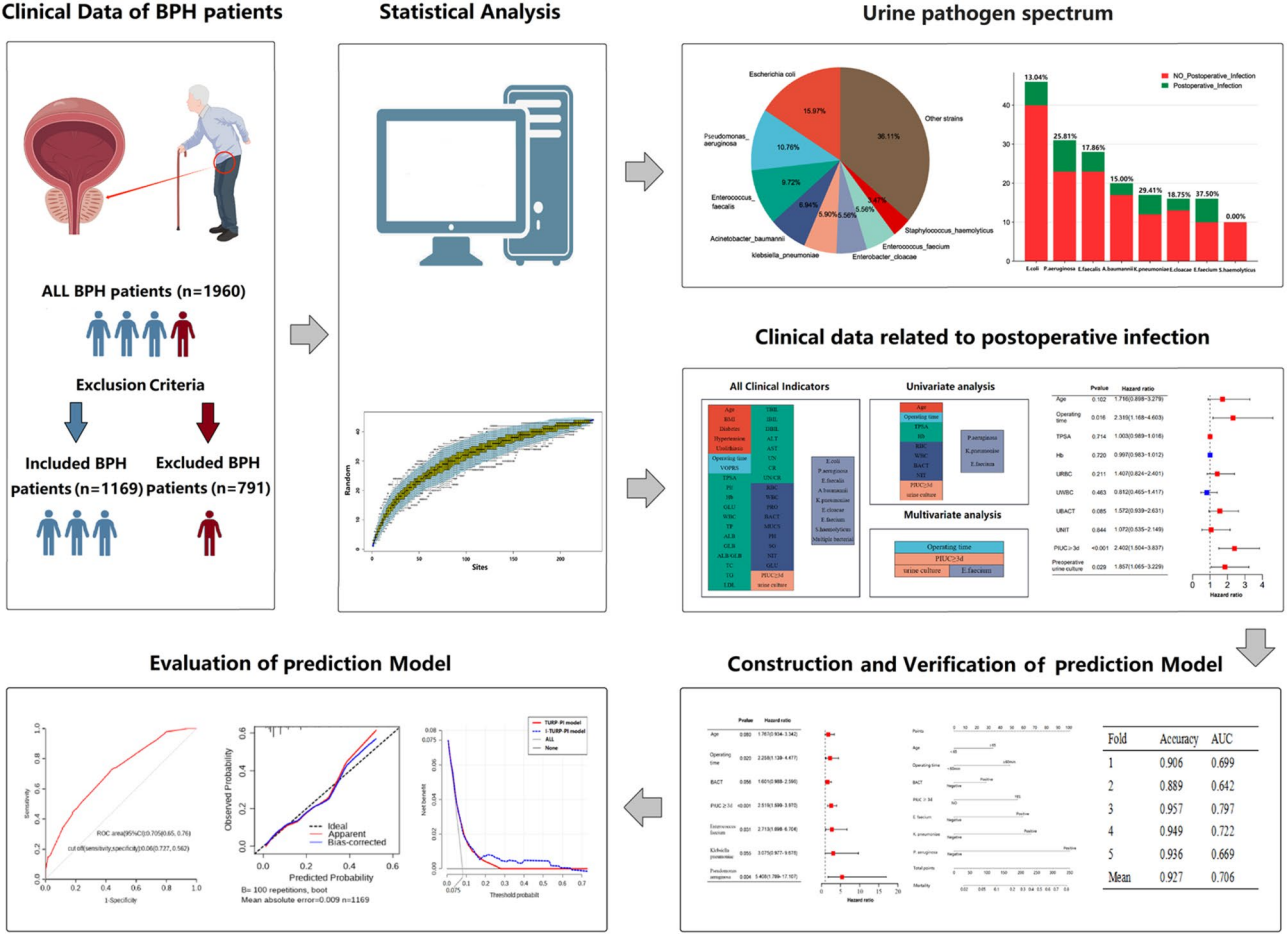
The wokflow of the study.

The workflow is shown in Fig. 1. This study's median age was 70 (65, 75) years old, and the median BMI was 23.14 (21.22, 25.05) kg/m^2^. Among 1169 patients, 188 (16.08%) got diabetes, 466 (39.86%) had hypertension, 157 (13.43%) had upper urinary tract stones, and 319 (27.29%) retained urinary catheters for more than three days before surgery. The median operation duration time was 87 (62, 117) min. The median removed volume of the prostate was 20.8 (9.8, 41.6) ml. The details are shown in Table S1.

### Risks for postoperative infection

Univariate analysis showed that postoperative infection was related to age, operation duration, preoperative total prostate-specific antigen (TPSA), preoperative hemoglobin (Hb), preoperative urine red blood cell microscopy (URBC), preoperative urine white blood cell microscopy (UWBC), preoperative urine bacteria (UBACT) were quantitative, preoperative urine nitrite qualitative, PIUC and positive preoperative urine culture (all *P* < 0.05, Tables 1, S2–6). Operation duration, indwelling catheter, and positive preoperative urine culture pre-surgery were independent risk factors for post-surgery infection for BHP patients (all *P* < 0.05, Table 1). The coefficient of each risk factor is shown in Fig. S1.

**Table 1:** Univariate and multivariate analysis of risk factors for infection after TURP.

Parameters		Non- infection n = 1081	Infection n = 88	*P*	
				Univariate	Multivariate
Age, years	< 65	271	12	0.016*	–
	≥ 65	810	76		
Operation time,min	< 60	269	10	0.004**	0.016*
	≥ 60	812	78		
TPSA	ng/mL	5.26 (2.67, 8.88)	6.37 (3.13, 11.22)	0.024*	–
Hb	g/L	144 (133, 151)	140 (130, 148)	0.043*	–
URBC	≤ 3/HP	486	25	0.003**	–
	> 3/HP	595	63		
UWBC	≤ 5/HP	689	42	0.003**	–
	> 5/HP	392	46		
UBACT	≤ 94/μL	548	29	0.001**	–
	> 94/μL	533	59		
UNIT	Negative	975	16	0.004**	–
	Positive	986	72		
PIUC	< 3d	805	45	< 0.001***	< 0.001***
	≥ 3d	276	43		
Urine culture	Negative	880	54	< 0.001***	0.029*
	Positive	201	34		

*P < 0.05, **P < 0.01, ***P < 0.001; TPSA: total prostate-specific antigen, Hb: hemoglobin, URBC: urine red blood cell, UWBC: urine white blood cell, UBACT: urine bacteria, UNIT: urine nitrite, PIUC: preoperative indwelling urinary catheter.

### Urine pahtogen spectrum

**Figure 2:**
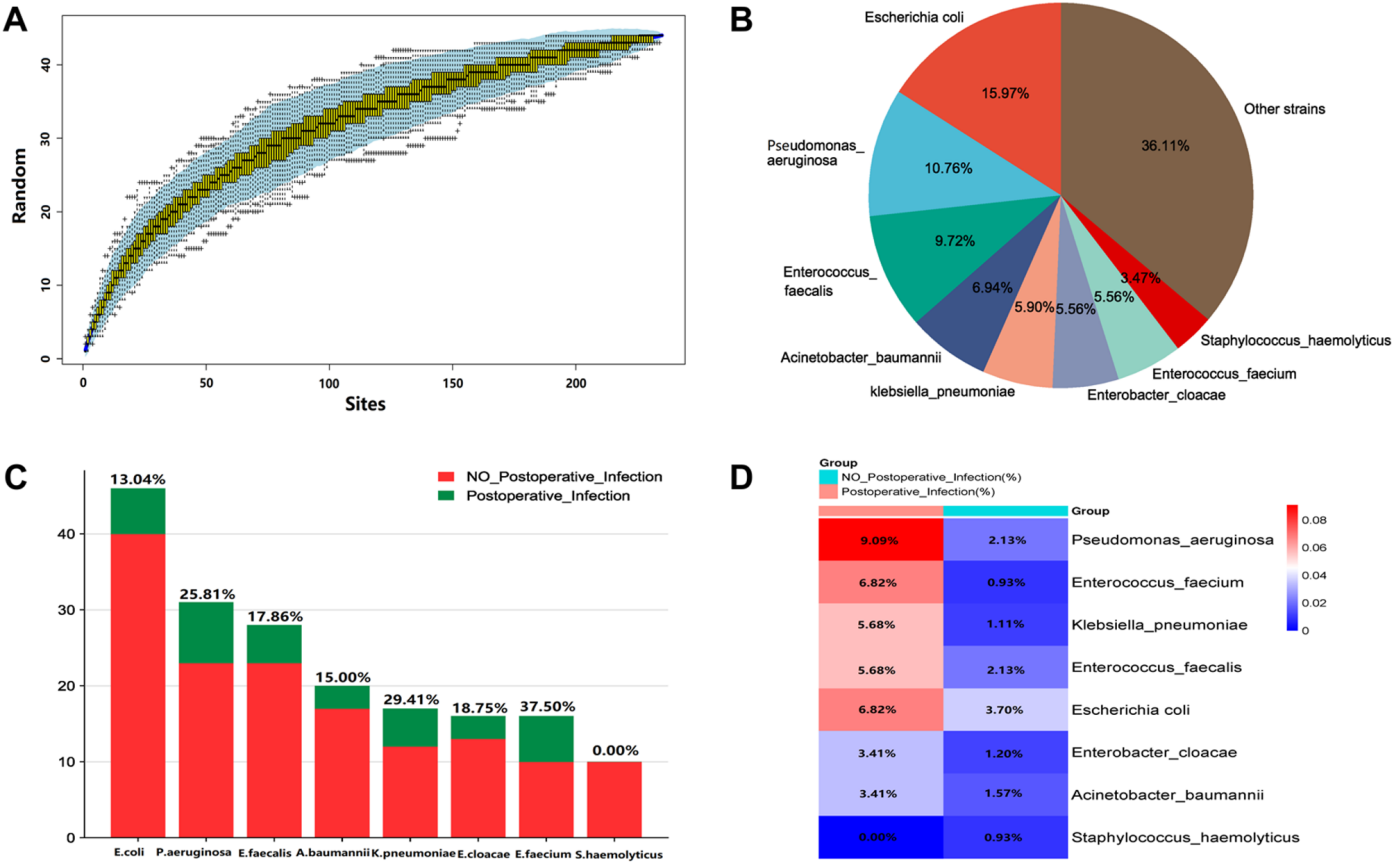
Distribution of urine pathogen. (**A**) Species accumulation curve. (**B**) Proportion of different pathogens in preoperative urine culture. (**C**) Incidence of postoperative infection in different pathogen-positive groups. (**D**) Comparison of detection rates of different pathogens in non_postoperative and postoperative infection groups.

235 (20.10%) patients had positive urine culture before the operation, 88 (7.53%) patients developed infections after TURP, and 44 (3.76%) had multiple infections. The species accumulation curve indicated that the sampling was sufficient for data analysis (Fig. 2A). The most common pathogens were *Escherichia coli* (15.97%), followed by *Pseudomonas aeruginosa* (10.76%) and *Enterococcus faecalis* (9.72%) (Fig. 2B, Table 2). Furthermore, *Enterococcus faecium* was an independent risk factor for postoperative infection by multivariate analysis (*P* = 0.014). The details are showed in Fig. 2C,D and Table 2.

**Table 2:** Species of urine cultured pathogen and related statistical analysis results.

Species		Non-infection = 1081	Infection n = 88	χ^2^	*P*	
					Univariate	Multivariate
*Escherichia coli*	Negative	1041	82	1.349	0.245	–
	Positive	40	6			
*Pseudomonas aeruginosa*	Negative	1058	80	12.706	< 0.001***	0.086
	Positive	23	8			
*Enterococcus faecalis*	Negative	1058	83	3.008	0.083	–
	Positive	23	5			
*Acinetobacter baumannii*	Negative	1064	85	0.723	0.395	–
	Positive	17	3			
*Klebsiella pneumoniae*	Negative	1069	83	8.892	0.003**	0.123
	Positive	12	5			
*Enterobacter cloacae*	Negative	1068	85	1.528	0.216	–
	Positive	13	3			
*Enterococcus faecium*	Negative	1071	82	16.797	< 0.001***	0.014*
	Positive	10	6			
*Staphylococcus haemolyticus*	Negative	1071	88	–	1.000	–
	Positive	10	0			
Multiple bacterials	Negative	1044	81	3.447	0.063	–
	Positive	37	7			

*P < 0.05, **P < 0.01, ***P < 0.001; Pathogen species detected ≥ 10 times were calculated.

### Predictive model construction and validation

**Figure 3:**
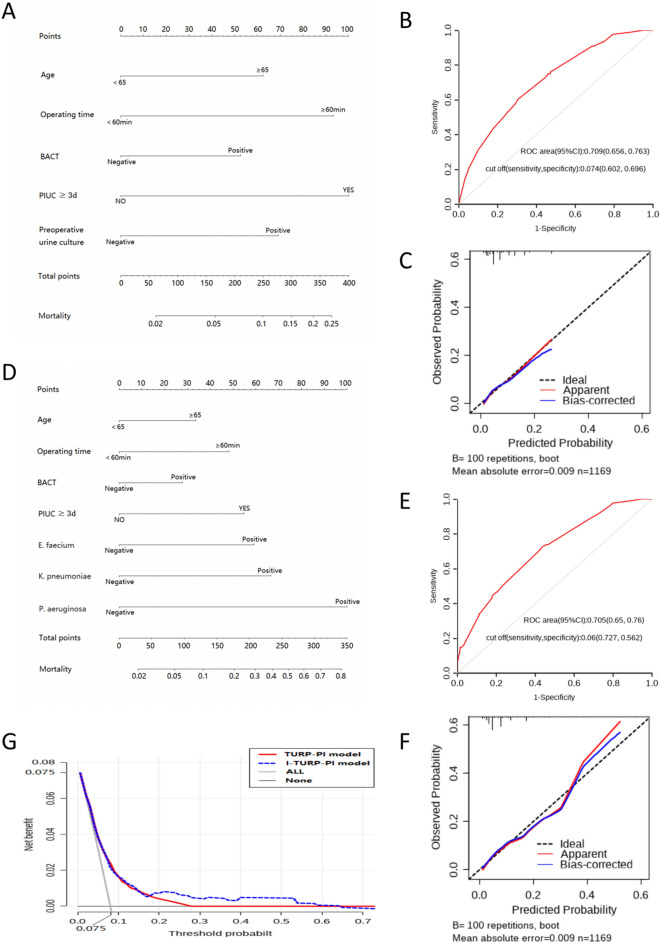
Nomogram for two predictive models. (**A**) TURP-PI model; (**B**) ROC of TURP-PI model; (**C**) The calibration curve of TURP-PI model; (**D**) I-TURP-PI model; (**E**) The ROC of I-TURP-PI model; (**F**) The calibration curve of I-TURP-PI model; (**G**) The clinical impact curves of two models.

Risk factors to build the TURP-postoperative infection (TURP-PI) and improved-TURP-PI (I-TURP-PI) models were shown in Table S7. Each factor's coefficient is shown in Fig. S2. The nomogram exhibited the models’ application (Fig. 3A,D). The two models were stable and credible by fivefold cross-validation (Table S8). The area under the curve (AUC) of the two models were above 0.7 (AUC_TURP-PI_ = 0.709 (95% CI 0.656–0.763), AUC_I-TURP-PI_ = 0.705 (95% CI 0.650–0.760) (Fig. 3B,E). Then, calibration curves showed they were reliable by the Hosmer–Lemeshow test (*P*_TURP-PI_ = 0.983, *P*_I-TURP-PI_ = 0.988). Also, the decision curves showed the superiority of the I-TURP-PI model based on the net benefit and threshold probabilities (1.2–62.7%), compared to the TURP-PI model (1.8–26.3%) (Fig. 3C,F), indicating I-TURP-PI models could benefit more extensive patients. (Fig. 3G).

## Discussion

TURP has been the standard golden treatment for BPH patients for over a century since its invention ^14^. Our previous study showed that, for patients with upper urinary tract stones, bacterial species of preoperative urine culture impact the post-surgery infection after percutaneous nephrolithotomy (PCNL) ^15^. However, as far as we know, its role in infection prediction of patients with TURP hasn't been studied in the literature. We found that specified types of infected pathogens in urine before surgery can influence the postoperative infection rate of TURP.

In our study, we found patients with positive preoperative urine culture were more than 2.5 times likelihood to develop postoperative infections, compared with patients with negative one (14.47% VS 5.78%). Patients infected with *Enterococcus faecium* were most susceptible to infection after TRUP, followed by *Klebsiella pneumoniae* and *Pseudomonas aeruginosa*. These three pathogens were also reported to be responsible for secondary bacteremia in children with symptomatic nosocomial urinary tract infections ^16^. By reading the extensive literature, we found that all three bacteria have their own characteristics. Enterococcus faecium is resistant to many antimicrobial agents and could cause serious incidence rates and mortality ^17–19^. *Klebsiella pneumoniae* has strong hidden abilities and invasiveness, leading to immune escape and resistance to urine erosion ^20,21^. It could cause urinary tract infections, pneumonia, liver abscess, surgical site infections, and bloodstream infections ^22^. Pseudomonas aeruginosa is an antibiotic-resistant pathogen of great concern to the World Health Organization (WHO)^23^. It has many resistance mechanisms, such as antibiotic-modifying enzymes, obtaining plasmids encoding drug-resistance genes, limited penetration of antibiotics, and the possibility of generating energy-dependent pumps ^24^. Elderly patients infected by Pseudomonas aeruginosa could even relapse after antibiotic treatment ^25^. Understanding the characteristics of these bacteria could help clinicians pay more attention to specific bacteria in preoperative urine culture to better infection prevention and rational use of antibiotics.

Our research shows that some species are commonly detected in urinary tract infections, such as *Escherichia coli* and *Enterococcus faecalis*. But they don't have the solid pathogenicity to induce infection after surgery. While for some specific species, even with strong antibiotics pre-surgery, patients are still prone to get infections^9^. There are two main reasons for this confusion phenomenon. For bacteria, some certain kinds are easily cultured and resistant to drugs, leading a strong pathogenicity ^19,26^. They “escape” from surveillance of antibiotics in many ways, such as cell stress responses, biofilm formation, and genetic material changes^22,24,27^. For the host, BPH usually happens in older men with poor self-care abilities and the weakened immune system. Preoperative positive urine culture with certain bacteria in BPH patients should be taken seriously to reduce the infection after surgery.

Besides positive preoperative urine culture, operation time and PIUC were also independent risk factors for infection after TURP, which is in accordance with previous studies ^28–30^. The application of PICU could not only induce biofilm formation but also impair the defense mechanisms of the urinary tract, making patients susceptible to infection ^31^. A previous study also confirmed once organisms gained access to the catheterized urinary tract, low-level bacteriuria could rapidly progress to high-level in 24–48 h ^32^.

We established two models with many influence clinical parameters to optimize their prediction power. Although the two models had similar prediction abilities and model fitting degrees, the I-TURP-PI model could benefit more BPH patients than the TURP-PI model. Most importantly, when constructing the I-TURP-PI model, we excluded “urine culture” from multivariate analysis. Instead, pathogens species in urine culture were then included in the model construction, indicating specific pathogens in urine were the key factors affecting postoperative infection.

The highlight of our research is that, for the first time, we found the specific types of pathogens in preoperative urine could increase the infection probability after TURP. We then used the pathogen species as the evaluation index for model-building to predict infection after TURP. In clinical practice, it often takes 2–3 days to obtain a post-operative urine culture result, leading to untimely administration of appropriate antibiotics. Thus, advance knowledge of common urinary tract bacteria and their corresponding characteristics of infection could be of great help in clinical diagnosis and treatment.

However, there were some limitations. Firstly, our study was a retrospective and one-center design. The data might be biased. Prospective clinical trials with more extensive patients were needed. Secondly, the antibiotic resistance of pathogens varies in different regions, which may lead to some deviations. However, we can map the species' spectrum in the various areas for proper antibiotic treatment. Thirdly, data such as Free-PSA and types of preoperative antibiotics, which may influence the incidence of postoperative infections, were not included in our model because they were unavailable.

## Conclusion

The positive preoperative urine culture of Enterococcus faecium, Klebsiella pneumoniae, and Pseudomonas aeruginosa, could affect the postoperative infection of BPH patients with TURP. Our models might help predict the occurrence of postoperative infection and guide the use of antibiotics in clinical settings. Clinicians could focus more on pathogens species in preoperative urine cultures when making clinical decisions for patients undergoing TURP.

## Materials and methods

### Study population and data collection

Our study was a single-center and case–control design, approved by the Institutional Review Board of the Fujian Provincial Hospital Ethics Committee, in compliance with the Declaration of Helsinki (K2023-01-011). From January 2015 to June 2022, 1960 BPH patients who underwent TURP in the urology department of Fujian provincial hospital were enrolled. All BPH patients received prophylactic antibiotics during the perioperative period. We included patients whose urine cultures were initially positive but turned negative after antibiotic treatment. And diabetes patients whose fasting plasma glucose was controlled below eight mmol/L. Patients with bladder stones or tumors, blood system diseases, immune system diseases, other system infections (< 1 mouth), patients undergoing other operations during the same period, and patients without complete clinical information were excluded. Finally, 1169 patients were included in our study. The patient selection is shown in Fig. S3.

The following data were collected: (1) preoperative information: age, BMI, complications, laboratory tests. (2) operative information: operating time and volume of surgically removed prostate. (3) postoperative information such as maximum temperature and blood culture.

### Criteria of infection

Postoperative infection was defined as positive blood culture or body temperature above 38.5℃. Other systemic infections or unknown fevers were excluded.

### Risks for postoperative infection

Thirty-eight clinical parameters were included for univariate analysis, such as indwelling catheter before surgery, the preoperative urine culture, and operating time. Then multivariate analysis was performed to identify independent risk factors that could affect post-surgery infection.

### Urine pathogen spectrum

We performed univariate analysis to analyze the relationship between pathogen species and infection. Species accumulation curves were plotted.

### Identification and validation of the predictive models

We first used the risk factors which significantly affect postoperative infection by univariate analysis to construct a predictive model (TURP-PI model). Then "pathogen species" was added to the TURP-PI model to create an improved-TURP-PI model (I-TURP-PI) to see whether pathogen species could enhance the prediction ability. Furthermore, the two models were compared and assessed by receiver operator characteristic curve (ROC) curves, the Hosmer–Lemeshow test, and decision curve analysis. They were also validated by five-fold cross-validation.

### Risk score

We calculated each patient's risk score based on the formula below: Risk score _TURP-PI_ = − 4.451 + (0.567) × (age) + (0.845) × (operation time) + (0.477) × (UBACT) + (0.905) × (PIUC ≥ 3d) + (0.626) × (Preoperative urine culture), Risk score _I-TURP-PI_ = − 0.570 + (0.569) × (age) + (0.814) × (operation time) + (0.471) × (UBACT) + (0.924) × (PIUC ≥ 3d) + (0.998) × (*Enterococcus faecium*) + (1.123) × (*Klebsiella pneumoniae*) + (1.688) × (*Pseudomonas aeruginosa*).

### Statistical analysis

SPSS (16.0 version) was used to analyze data. The t-test, a rank-sum test, chi-square test, and logistic regression were performed to analyze data. *P* < 0.05 was statistically significant. Visualization was performed through R software (version 3.6.1).

### Ethical approval

Our study was approved by the Institutional Review Board of the Fujian Provincial Hospital Ethics Committee, in compliance with the Declaration of Helsinki (K2023-01-011).

### Consent to participate

This study was retrospective and approved by the Ethics Committee of Fujian Provincial Hospital. The exemption from the Informed Consent Form was approved by the Ethics Committee of Fujian Provincial Hospital.

### Supplementary Information

Supplementary Figures.

Supplementary Figure S3.

Supplementary Tables.

## Data Availability

The datasets generated during and/or analyzed during the current study are available from the corresponding author on reasonable request, provided that no copy rights are violated.

## Abbreviations

BPH: Benign prostatic hyperplasia
TURP: Transurethral resection of the prostate
PIUC: Preoperative indwelling urinary catheter
PSA: Prostate-specific antigen
IQR: Interquartile range
TPSA: Prostate-specific antigen
PLT: Platelet
Hb: Hemoglobin
GLU: Glucose
WBC: White blood cell
TP: Total protein
ALB: Albumin
GLB: Globulin
ALB/GLB: Albumin/globulin
TC: Total cholesterol
TG: Triglyceride
LDL: Low-density lipoprotein
TBIL: Total bilirubin
IBIL: Indirect bilirubin
DBIL: Direct bilirubin
ALT: Alanine aminotransferase
AST: Aspartate transaminase
UN: Urea nitrogen
CR: Creatinine
UN/CR: Urea nitrogen/creatinine
URBC: Urine red blood cell
UWBC: Urine white blood cell
UBACT: Urine bacteria
UNIT: Urine nitrite
PCNL: Percutaneous nephrolithotomy
ROC: Receiver operator characteristic curve
WHO: World Health Organization
