# Supplementary material for: Pathogen species are the risk factors for postoperative infection of patients with transurethral resection of the prostate: a retrospective study
Source: Sci Rep. 2023 Nov 28;13:20943. doi: 10.1038/s41598-023-47773-7 (PMC10684857; doi:10.1038/s41598-023-47773-7)
Supplement: Supplementary file 1 — Supplementary Figures. [file 41598_2023_47773_MOESM1_ESM.docx]

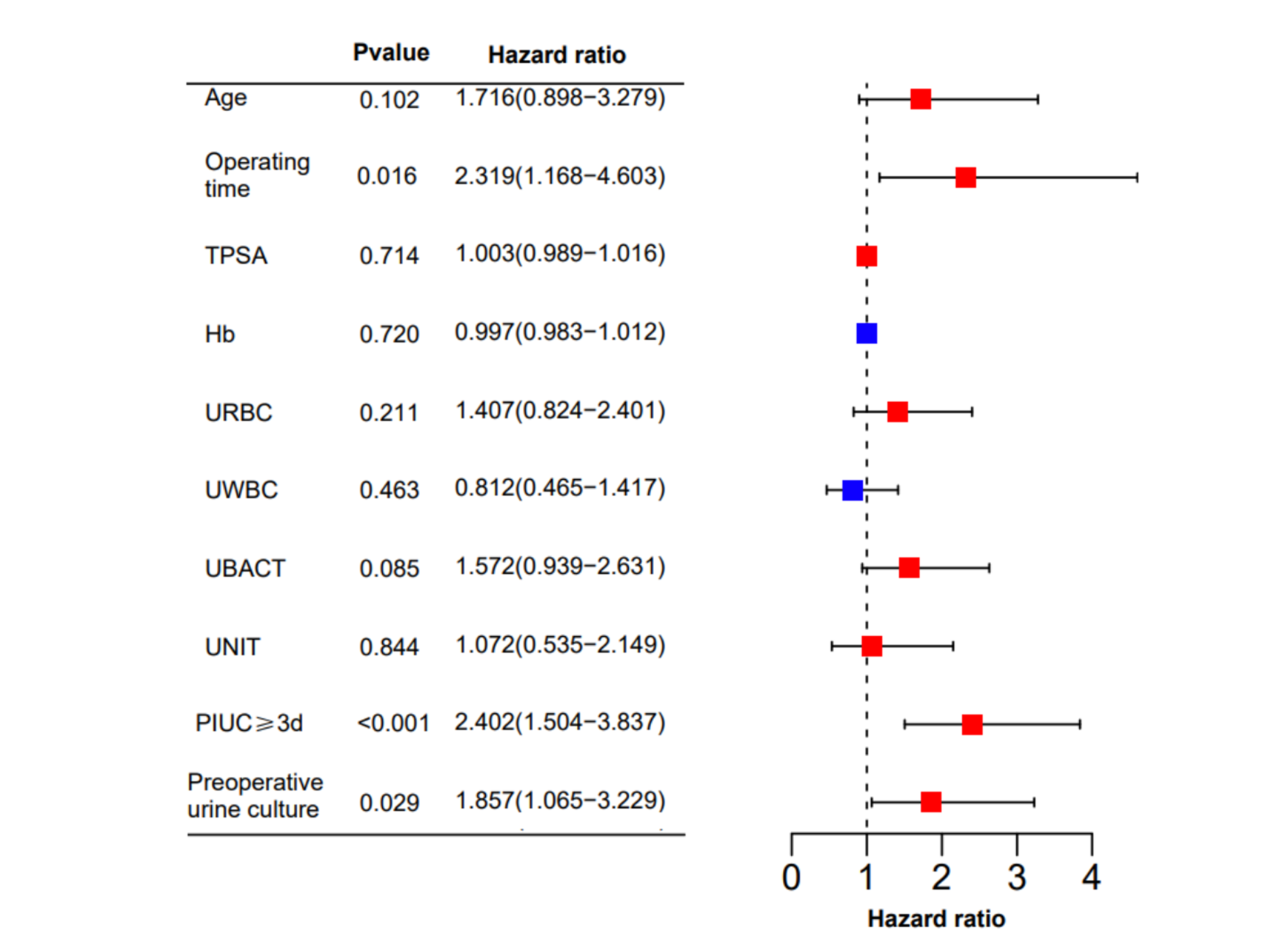


Figure S1 The coefficient of each risk factor.


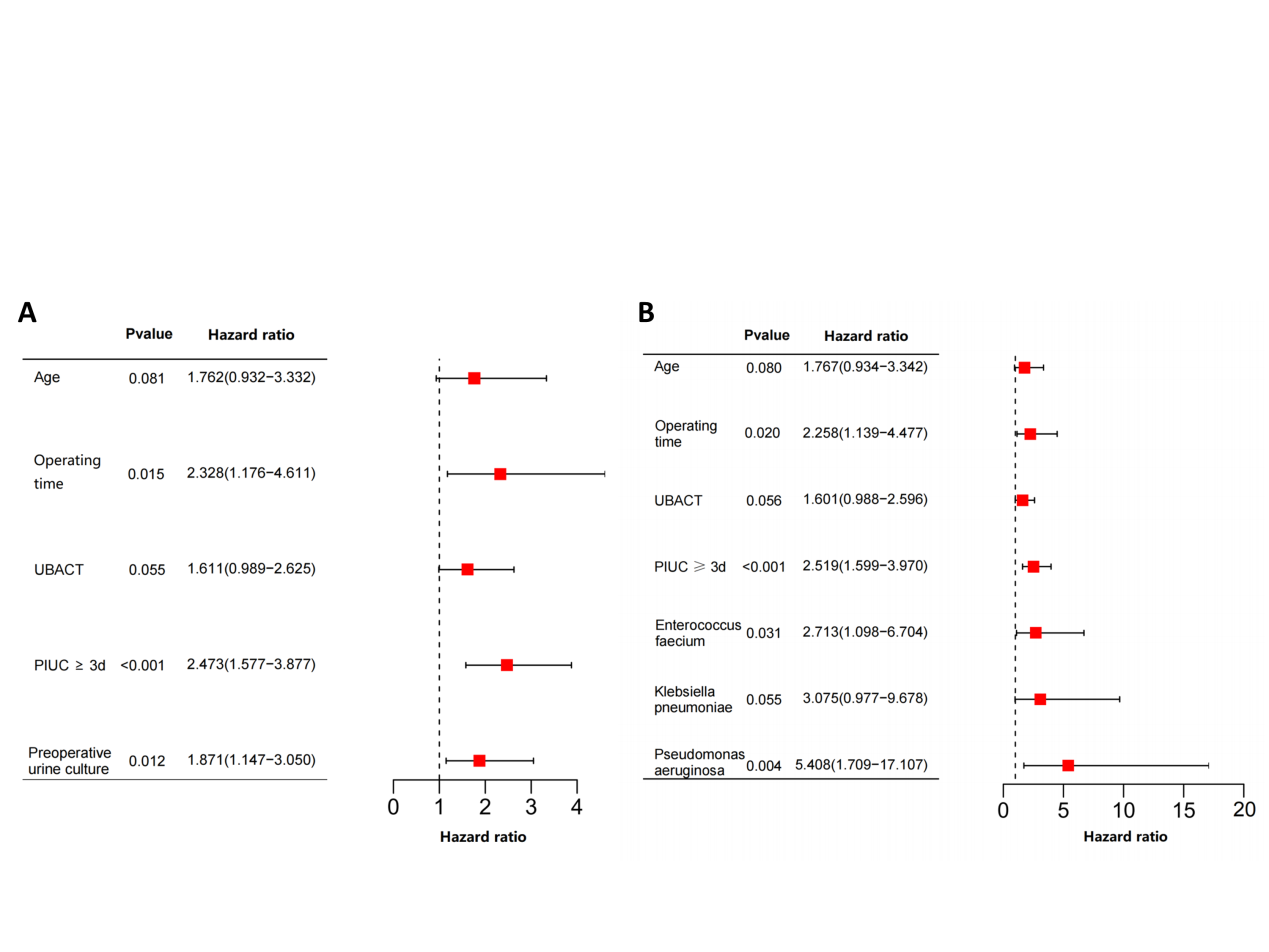


Figure S2 Each factor's coefficient of the two models.
