# Supplementary figures and images for: Pathogen species are the risk factors for postoperative infection of patients with transurethral resection of the prostate: a retrospective study
Source: Sci Rep. 2023 Nov 28;13:20943. doi: 10.1038/s41598-023-47773-7 (PMC10684857; doi:10.1038/s41598-023-47773-7)

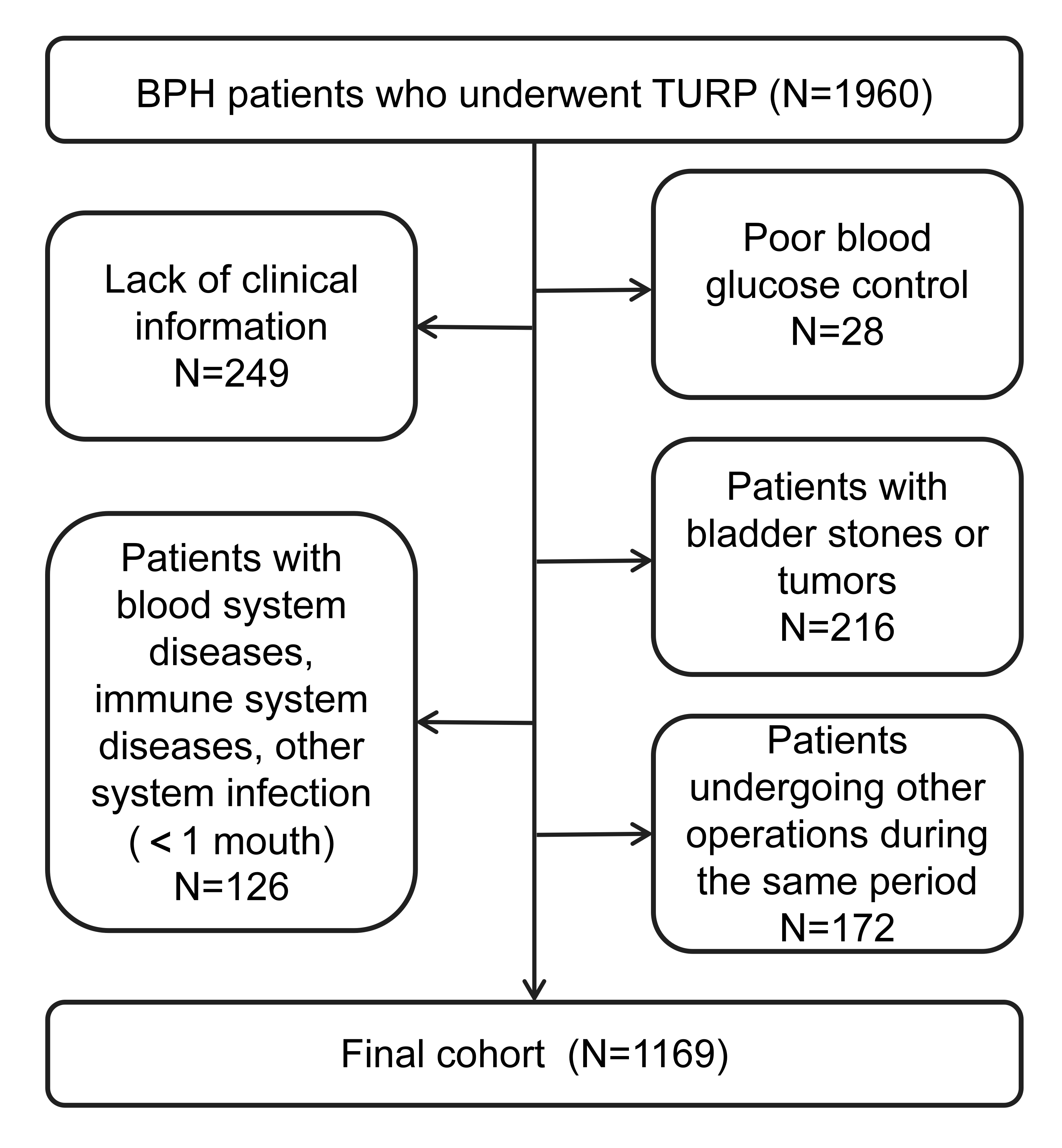


Figure S3 Patient selection.

Supplement: Supplementary file 2 — Supplementary Figure S3. [file 41598_2023_47773_MOESM2_ESM.docx]
