# Supplementary material for: Pathogen species are the risk factors for postoperative infection of patients with transurethral resection of the prostate: a retrospective study
Source: Sci Rep. 2023 Nov 28;13:20943. doi: 10.1038/s41598-023-47773-7 (PMC10684857; doi:10.1038/s41598-023-47773-7)
Supplement: Supplementary file 3 — Supplementary Tables. [file 41598_2023_47773_MOESM3_ESM.docx]

Table S1 Demographic details and baseline

|  | Total  n=1196 | Non- infection  n=1081 | Infection  n=88 |
| --- | --- | --- | --- |
| Age, years (IQR) | 70 (65-75) | 70 (64-75) | 71 (67-77) |
| BMI, kg/m2 (IQR) | 23.14 (21.22-25.05) | 23.12 (21.22-25.05) | 23.52 (21.31-25.16) |
| Diabetes, n (%) | 188 (16.08) | 170 (15.73) | 18 (20.45) |
| Hypertension, n (%) | 466 (39.86) | 430 (39.78) | 36 (40.91) |
| Urolithiasis, n (%) | 157 (13.43) | 142 (13.14) | 15 (17.05) |
| PIUC ≥ 3d, n (%) | 319 (27.29) | 276 (25.53) | 43 (48.86) |
| Operation time, min (IQR) | 87 (62-117) | 87 (61-117) | 90.5 (71.5-118) |
| Volume of removed prostate, ml (IQR) | 20.8 (9.8-41.6) | 20.8 (10.3-41.6) | 21.8 (9.4-44.2) |

IQR: interquartile range, PIUC: preoperative indwelling urinary catheter.

Table S2 Comparison of clinical characteristics between non_postoperative and postoperative infection groups.

|  |  | Non- infection  n=1081 | Infection  n=88 | χ² /z | *P* |
| --- | --- | --- | --- | --- | --- |
| Age, years | <65 | 271 | 12 | 5.797 | 0.016^*^ |
|  | ≥65 | 810 | 76 |  |  |
| BMI, kg/㎡ | ≤24 | 675 | 51 | 0.696 | 0.404 |
|  | >24 | 406 | 37 |  |  |
| Diabetes | N | 911 | 70 | 1.348 | 0.246 |
|  | Y | 170 | 18 |  |  |
| Hypertension | N | 651 | 52 | 0.043 | 0.835 |
|  | Y | 430 | 36 |  |  |
| Urolithiasis | N | 939 | 73 | 1.070 | 0.301 |
|  | Y | 142 | 15 |  |  |
| Operation time, min | <60 | 269 | 10 | 8.187 | 0.004^**^ |
|  | ≥60 | 812 | 78 |  |  |
| Volume of prostate removed surgically, ml | - | 20.8  (10.3,41.6) | 21.8  (9.4,46.05) | 0.300 | 0.764 |

*: *P* < 0.05, **: *P* < 0.01, ***: *P* < 0.001

Table S3 Comparison of preoperative blood indicators

|  |  | Non-infection n=1081 | Infection  n=88 | z | *P* |
| --- | --- | --- | --- | --- | --- |
| TPSA | ng/mL | 5.26  (2.67,8.88) | 6.37  (3.13,11.22) | 2.251 | 0.024^*^ |
| Plt | 10^9/L | 217  (182,261) | 216.5  (171,252.5) | 1.165 | 0.244 |
| Hb | g/L | 144  (133,151) | 140  (130,148) | 2.024 | 0.043^*^ |
| GLU | Mmol/L | 5.41  (5.06,5.98) | 5.42  (4.95,6.21) | 0.260 | 0.795 |
| WBC | 10^9/L | 6.22  (5.30,7.40) | 6.45  (4.84,7.98) | 0.571 | 0.568 |
| TP | g/L | 72.0  (68.30,75.30) | 72.45  (68.05,76.35) | 0.295 | 0.768 |
| ALB | g/L | 44  (41.00,46.00) | 43  (39.75,46.05) | 1.398 | 0.162 |
| GLB | g/L | 28  (26.00,31.00) | 28.95  (26.45,31.03) | 1.383 | 0.167 |
| ALB/GLB | - | 1.54  (1.38,1.72) | 1.50  (1.37,1.61) | 1.873 | 0.061 |
| TC | mmol/l | 4.64  (4.00,5.33) | 4.54  (3.87,5.35) | 0.378 | 0.705 |
| TG | mmol/l | 1.17  (0.86,1.61) | 1.11  (0.86,1.59) | 0.562 | 0.574 |
| LDL | mmol/l | 3.03  (2.51,3.64) | 3.03  (2.45,3.57) | 0.293 | 0.769 |
| TBIL | umol/l | 11.30  (8.40,15.10) | 12.02  (8.28,15.42) | 0.322 | 0.747 |
| IBIL | umol/l | 6.64  (4.60,9.10) | 6.69  (4.45,9.83) | 0.200 | 0.841 |
| DBIL | umol/l | 4.61  (3.53,5.99) | 4.71  (3.45,6.08) | 0.146 | 0.884 |
| ALT | U/L | 18.70  (14.00,26.00) | 20.00  (13.00,30.00) | 1.240 | 0.215 |
| AST | U/L | 20.00  (17.00,24.00) | 21.00  (17.00,26.00) | 1.891 | 0.059 |
| UN | mmol/l | 5.21  (4.30,6.31) | 5.10  (4.37,6.77) | 0.230 | 0.818 |
| CR | mmol/l | 79.95  (70.20,91.11) | 81.87  (70.25,102.62) | 1.325 | 0.185 |
| UN/CR | % | 6.36  (5.33,7.65) | 6.00  (5.03,7.05) | 1.676 | 0.094 |

*: *P* < 0.05, **: *P* < 0.01, ***: *P* < 0.001; TPSA: total prostate-specific antigen, PLT: platelet, Hb: hemoglobin, GLU: glucose, WBC: white blood cell, TP: total protein, ALB: albumin, GLB: globulin, ALB/GLB: albumin/globulin, TC: total cholesterol, TG: triglyceride, LDL: low-density lipoprotein, TBIL: total bilirubin, IBIL: indirect bilirubin, DBIL: direct bilirubin, ALT: alanine aminotransferase, AST: aspartate transaminase, UN: urea nitrogen, CR: creatinine, UN/CR: urea nitrogen/creatinine.

Table S4 Comparison of preoperative urine indicators

|  |  | Non- infection  n=1081 | Infection  n=88 | χ² /z | *P* |
| --- | --- | --- | --- | --- | --- |
| RBC | ≤3 | 486 | 25 | 9.058 | 0.003^**^ |
|  | >3 | 595 | 63 |  |  |
| WBC | ≤5 | 689 | 42 | 8.903 | 0.003^**^ |
|  | >5 | 392 | 46 |  |  |
| PRO | Negative, Trace | 860 | 71 | 0.064 | 0.801 |
|  | 1+、2+、3+ | 221 | 17 |  |  |
| BACT | ≤94 | 548 | 29 | 10.245 | 0.001^**^ |
|  | >94 | 533 | 59 |  |  |
| MUCS | /μl | 0.15  (0,0.95) | 0.20  (0,0.77) | 0.521 | 0.602 |
| PH | - | 6.0  (5.5,6.5) | 6.5  (5.5,7.0) | 0.480 | 0.631 |
| SG | - | 1.015  (1.012,1.020) | 1.014  (1.011,1.018) | 1.652 | 0.099 |
| NIT | Negative | 975 | 16 | 8.356 | 0.004^*^ |
|  | Positive | 986 | 72 |  |  |
| GLU | Negative | 1029 | 81 | 1.087 | 0.297 |
|  | Positive | 52 | 7 |  |  |

*: *P* < 0.05, **: *P* < 0.01, ***: *P* < 0.001; RBC: red blood cell, WBC: white blood cell, PRO: protein, BACT: bacterial count, MUCS: mucus filament, PH: the potential of hydrogen, SG: specific gravity, NIT: nitrite, GLU: glucose.

Table S5 Comparison of PIUC and urine culture

|  |  | Non-infection  n=1081 | Infection  n=88 | χ² | *P* |
| --- | --- | --- | --- | --- | --- |
| PIUC ≥3d | N | 805 | 45 | 22.326 | <0.001^***^ |
|  | Y | 276 | 43 |  |  |
| urine culture | Negative | 880 | 54 | 20.352 | <0.001^***^ |
|  | Positive | 201 | 34 |  |  |

*: *P* < 0.05, **: *P* < 0.01, ***: *P* < 0.001; PIUC: preoperative indwelling urinary catheter.

Table S6 Multivariate analysis results of risk factors for infection after TURP.

|  | B | SE | Wald | OR (95%CI) | *P* |
| --- | --- | --- | --- | --- | --- |
| Age | 0.540 | 0.330 | 2.669 | 1.716(0.898,3.279) | 0.102 |
| Operating time | 0.841 | 0.350 | 5.775 | 2.319(1.168,4.603) | 0.016^*^ |
| TPSA | 0.003 | 0.007 | 0.134 | 1.003(0.989,1.016) | 0.714 |
| Hb | 0.003 | 0.008 | 0.129 | 0.997(0.983,1.012) | 0.720 |
| URBC | 0.341 | 0.273 | 1.564 | 1.407(0.824,2.401) | 0.211 |
| UWBC | 0.209 | 0.284 | 0.538 | 0.812(0.465,1.417) | 0.463 |
| UBACT | 0.452 | 0.263 | 2.961 | 1.572(0.939,2.631) | 0.085 |
| UNIT | 0.070 | 0.355 | 0.039 | 1.072(0.535,2.149) | 0.844 |
| PIUC ≥ 3d | 0.876 | 0.239 | 13.453 | 2.402(1.504,3.837) | <0.001^***^ |
| Preoperative urine culture | 0.619 | 0.282 | 4.819 | 1.857(1.065,3.229) | 0.029^*^ |

*: *P* < 0.05, **: *P* < 0.01, ***: *P* < 0.001; TPSA: total prostate-specific antigen, Hb: hemoglobin, URBC: urine red blood cell, UWBC: urine white blood cell, UBACT: urine bacteria, UNIT: urine nitrite, PIUC: preoperative indwelling urinary catheter.

Table S7 The most significant variables included in the TURP-PI model and I-TURP-PI model.

|  |  | B | SE | Wald | OR (95%CI) | *p* |
| --- | --- | --- | --- | --- | --- | --- |
| TURP-PI model | Age | 0.567 | 0.325 | 1.743 | 1.762 (0.932,3.332) | 0.081 |
|  | Operating time | 0.845 | 0.349 | 2.424 | 2.328 (1.176,4.611) | 0.015 |
|  | UBACT | 0.477 | 0.249 | 1.916 | 1.611 (0.989,2.625) | 0.055 |
|  | PIUC ≥ 3d | 0.905 | 0.229 | 3.947 | 2.473 (1.577,3.877) | <0.001 |
|  | Preoperative urine culture | 0.626 | 0.249 | 2.511 | 1.871 (1.147,3.050) | 0.012 |
| I-TURP-PI model | Age | 0.569 | 0.325 | 1.751 | 1.767 (0.934,3.342) | 0.080 |
|  | Operating time | 0.814 | 0.349 | 2.332 | 2.258 (1.139,4.477) | 0.020 |
|  | UBACT | 0.471 | 0.246 | 1.911 | 1.601 (0.988,2.596) | 0.056 |
|  | PIUC ≥ 3d | 0.924 | 0.232 | 3.983 | 2.519 (1.599,3.970) | <0.001 |
|  | Enterococcus faecium | 0.998 | 0.462 | 2.162 | 2.713 (1.098,6.704) | 0.031 |
|  | Klebsiella pneumoniae | 1.123 | 0.585 | 1.920 | 3.075 (0.977,9.678) | 0.055 |
|  | Pseudomonas aeruginosa | 1.688 | 0.588 | 2.872 | 5.408 (1.709,17.107) | 0.004 |

*: *P* < 0.05, **: *P* < 0.01, ***: *P* < 0.001; UBACT: urine bacteria, PIUC: preoperative indwelling urinary catheter.

|  | Fold | Cox-Snell R2 | Nagelkerke R2 | Accuracy | AUC |
| --- | --- | --- | --- | --- | --- |
| TURP-PI model | 1 | 0.164 | 0.19 | 0.897 | 0.65 |
|  | 2 | 0.155 | 0.177 | 0.919 | 0.673 |
|  | 3 | 0.147 | 0.166 | 0.932 | 0.761 |
|  | 4 | 0.162 | 0.181 | 0.949 | 0.737 |
|  | 5 | 0.145 | 0.164 | 0.927 | 0.701 |
|  | Mean | 0.155 | 0.176 | 0.925 | 0.704 |
| I-TURP-PI model | 1 | 0.183 | 0.21 | 0.906 | 0.699 |
|  | 2 | 0.184 | 0.215 | 0.889 | 0.642 |
|  | 3 | 0.173 | 0.193 | 0.957 | 0.797 |
|  | 4 | 0.177 | 0.198 | 0.949 | 0.722 |
|  | 5 | 0.194 | 0.219 | 0.936 | 0.669 |
|  | Mean | 0.182 | 0.207 | 0.927 | 0.706 |

Table S8 Five-fold cross-validation of the TURP-PI and I- TURP-PI model.
